# Mining Clinical Notes for Physical Rehabilitation Exercise Information: Natural Language Processing Algorithm Development and Validation Study

**DOI:** 10.2196/52289

**Published:** 2024-04-03

**Authors:** Sonish Sivarajkumar, Fengyi Gao, Parker Denny, Bayan Aldhahwani, Shyam Visweswaran, Allyn Bove, Yanshan Wang

**Affiliations:** 1 Intelligent Systems Program University of Pittsburgh Pittsburgh, PA United States; 2 Department of Health Information Management University of Pittsburgh Pittsburgh, PA United States; 3 Department of Physical Therapy University of Pittsburgh Pittsburgh, PA United States; 4 Department of Physical Therapy Umm Al-Qura University Makkah Saudi Arabia; 5 Department of Biomedical Informatics University of Pittsburgh Pittsburgh, PA United States; 6 Clinical and Translational Science Institute University of Pittsburgh Pittsburgh, PA United States

**Keywords:** natural language processing, electronic health records, rehabilitation, physical exercise, ChatGPT, artificial intelligence, stroke, physical rehabilitation, rehabilitation therapy, exercise, machine learning

## Abstract

**Background:**

The rehabilitation of a patient who had a stroke requires precise, personalized treatment plans. Natural language processing (NLP) offers the potential to extract valuable exercise information from clinical notes, aiding in the development of more effective rehabilitation strategies.

**Objective:**

This study aims to develop and evaluate a variety of NLP algorithms to extract and categorize physical rehabilitation exercise information from the clinical notes of patients who had a stroke treated at the University of Pittsburgh Medical Center.

**Methods:**

A cohort of 13,605 patients diagnosed with stroke was identified, and their clinical notes containing rehabilitation therapy notes were retrieved. A comprehensive clinical ontology was created to represent various aspects of physical rehabilitation exercises. State-of-the-art NLP algorithms were then developed and compared, including rule-based, machine learning–based algorithms (support vector machine, logistic regression, gradient boosting, and AdaBoost) and large language model (LLM)–based algorithms (ChatGPT [OpenAI]). The study focused on key performance metrics, particularly *F*_1_-scores, to evaluate algorithm effectiveness.

**Results:**

The analysis was conducted on a data set comprising 23,724 notes with detailed demographic and clinical characteristics. The rule-based NLP algorithm demonstrated superior performance in most areas, particularly in detecting the “Right Side” location with an *F*_1_-score of 0.975, outperforming gradient boosting by 0.063. Gradient boosting excelled in “Lower Extremity” location detection (*F*_1_-score: 0.978), surpassing rule-based NLP by 0.023. It also showed notable performance in the “Passive Range of Motion” detection with an *F*_1_-score of 0.970, a 0.032 improvement over rule-based NLP. The rule-based algorithm efficiently handled “Duration,” “Sets,” and “Reps” with *F*_1_-scores up to 0.65. LLM-based NLP, particularly ChatGPT with few-shot prompts, achieved high recall but generally lower precision and *F*_1_-scores. However, it notably excelled in “Backward Plane” motion detection, achieving an *F*_1_-score of 0.846, surpassing the rule-based algorithm’s 0.720.

**Conclusions:**

The study successfully developed and evaluated multiple NLP algorithms, revealing the strengths and weaknesses of each in extracting physical rehabilitation exercise information from clinical notes. The detailed ontology and the robust performance of the rule-based and gradient boosting algorithms demonstrate significant potential for enhancing precision rehabilitation. These findings contribute to the ongoing efforts to integrate advanced NLP techniques into health care, moving toward predictive models that can recommend personalized rehabilitation treatments for optimal patient outcomes.

## Introduction

Precision medicine is a promising field of research that aims to provide personalized treatment plans for patients [1]. Recent years have seen a rise in interest in this field, as advances in machine learning and data collection techniques have greatly facilitated this research [2]. However, the principles of precision medicine have primarily been applied to the development of medications, and relatively little research has been conducted on their applications in other areas [3]. For instance, although rehabilitation clinics require individualized treatment procedures for patients, little research has been conducted on methods that use data analysis and machine learning to facilitate the design of such procedures [4]. Although the application of precision medicine to physical therapy has proven effective in improving the health of patients, current methods of creating personalized treatments rarely use automated approaches to facilitate decision support [5]. Thus, there is a need for tools to assist in the development of personalized treatments in physical therapy [6]. In the treatment of patients who had a stroke, the lack of decision support tools is especially pronounced, as the available treatments for this condition have not led to consistent outcomes across patient populations [7].

To develop decision support tools for the design of precision rehabilitation treatments for patients who had a stroke, it would be necessary to use electronic health record data to develop a predictive model of existing treatment options and their impact on patient outcomes [8]. However, physical therapy procedures are typically described in unstructured clinical notes, meaning that simple data extraction methods such as database queries cannot be applied to obtain sufficient information. Additionally, the language used to describe these procedures can differ between clinicians, locations, and periods [9]. More advanced natural language processing (NLP) algorithms are required to extract this information from clinical notes, but such a method has not yet been developed for this application.

In this paper, we aim to develop and evaluate NLP algorithms to extract physical rehabilitation exercise information from the clinical notes in the electronic health record. Our specific contributions are as follows. First, we created a novel and comprehensive clinical ontology to represent physical rehabilitation exercise information, which includes the type of motion, side of the body, location on the body, the plane of motion, duration, information on sets and reps, exercise purpose, exercise type, and body position. Second, we developed and compared a variety of NLP algorithms leveraging state-of-the-art techniques, including rule-based NLP algorithms, machine learning–based NLP algorithms (ie, support vector machine [SVM], logistic regression [LR], gradient boosting, and AdaBoost), and large language model (LLM)–based NLP algorithms (ie, ChatGPT [OpenAI] [10]) for the extraction of physical rehabilitation exercise from clinical notes. We are among the first to evaluate the capabilities of ChatGPT in extracting useful information from clinical notes.

## Methods

### Overview

**Figure 1:**
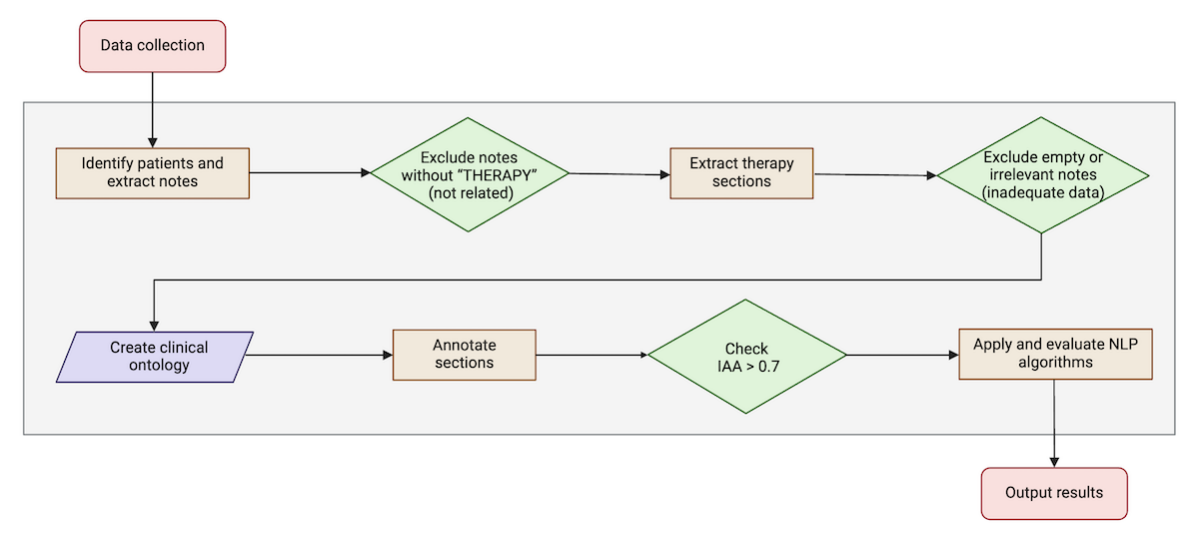
Flowchart illustrating the data flow throughout the study. IIA: interannotator agreement (IAA); NLP: natural language processing.

Figure 1 illustrates the data flow and the various stages of the research process. Each of these stages will be described in detail in the following sections.

### Data Collection

The study identified a cohort of patients diagnosed with stroke between January 1, 2016, and December 31, 2016, at University of Pittsburgh Medical Center (UPMC). For these patients, clinical encounter notes created between January 1, 2016, and December 31, 2018, were extracted from the institutional data warehouse. Table 1 provides the demographic characteristics of the patients included in this data set.

**Table 1:** Demographic information of patients included in the unfiltered data set (N=13,605).

Demographics		Values
Age (years), mean (SD)		75 (16)
**Gender, n (%)**		
	Female	6931 (51)
	Male	6673 (49)
**Race, n (%)**		
	Asian	64 (0.5)
	Black	1325 (9.7)
	White	11,661 (86)
	Other	153 (1.1)
	Not specified	402 (3)
**Ethnicity, n (%)**		
	Hispanic or Latinx	64 (0.5)
	Not Hispanic or Latinx	12,471 (92)
	Not specified	984 (7.2)

### Ethical Considerations

The study was approved by the University of Pittsburgh’s institutional review board (#21040204).

### Clinical Ontology for Physical Rehabilitation Exercise

To determine the relevance and hierarchy of extracted information, we developed a clinical ontology consisting of 9 categories of concepts relating to exercise descriptions, informed by consultation with clinical experts (PD, BA, and AB) in the field of physical therapy. In developing our clinical ontology, we also consulted established frameworks such as the International Classification of Functioning, Disability, and Health (ICF) [11] and the Systematized Nomenclature of Medicine—Clinical Terms (SNOMED CT) [12]. These comprehensive systems offered valuable insights into the structuring and categorization of health-related concepts, which we adapted for the specific context of physical rehabilitation exercises. Additionally, our ontology incorporates principles from the Unified Medical Language System (UMLS) [13] to ensure compatibility and interoperability with other health care informatics systems.

Each category was given a set of values, as well as examples of how those values might be expressed in clinical notes. The categories are type of motion, side of the body, location on the body, the plane of motion, duration, information on sets and reps, exercise purpose, exercise type, and body position. The ontology also includes examples of indications that the mentioned exercise was not performed during the visit corresponding to the clinical note. This ontology was used to inform both the structure of the annotations and the methods used to extract relevant documents from the data set.

The ontology reflects the complexity and nuance of physical rehabilitation exercises by incorporating terms and categories that are sensitive to the variations and specificities observed in clinical settings. This approach ensures that the ontology not only represents the theoretical model of rehabilitation exercises but also aligns with the practical, real-world application and documentation by health care professionals. Table 2 displays the 9 categories for 3 exercise descriptions (performed in-office, home exercise program, and not performed), with sets and reps split into separate rows and including negations and out-of-office exercises at the bottom.

**Table 2:** Summary of the clinical ontology used for annotations.

Category	Data type	Concepts
Exercise description	Enumerated	Performed in-office, home exercise program, not performed
Type of motion	Enumerated	ROM^a^, active ROM, active-assisted ROM, and passive ROM
Side of body	Enumerated	Right, left, bilateral, unilateral, contralateral, and ipsilateral
Location on body	Enumerated	Upper extremity (arms), lower extremity (legs), hip, thigh, knee, ankle, foot, heel, toe, shoulder, scapula, elbow, forearm, wrist, hand, thumb, head, neck, chest, abdomen, and lower back
Plane of motion	Enumerated	Flexion, extension, abduction, adduction, internal rotation, external rotation, lateral flexion, horizontal abduction, horizontal adduction, protraction, retraction, elevation, depression, inversion, eversion, pronation, supination, plantarflexion, dorsiflexion, radial deviation, ulnar deviation, upward rotation, downward rotation, opposition, forward, backward, lateral, medial, scaption, rotation, closure, clockwise, counterclockwise, distraction, all planes, anterior, posterior, horizontal, vertical, diagonal, and gravity elimination
Duration (seconds)	Integer	N/A^b^
Number of sets	Integer	N/A
Number of reps	Integer	N/A
Exercise purpose	Enumerated	Strength, fine motor, motor control, perception, simulated, power, endurance, joint mobility, joint alignment, muscle flexibility, cardio, pulmonary, agility, and vestibular
Exercise type	Enumerated	Upper extremity strength, lower extremity strength, trunk or core strength, scapular strength, ROM, flexibility or mobility, balance or vestibular, gait training, cardio or aerobic, and functional mobility
Body position	Binary	Weight bearing and non-weight bearing
Negation or hypothetical	Binary	Held or not performed and home exercise program

^a^ROM: range of motion.

^b^N/A: not applicable.

### Preprocessing and Section Extraction

Physical therapeutic procedures were usually documented in the section “THERAPY.” Therefore, we first filtered out the notes that did not contain a physical therapy visit by excluding files whose names lacked the string “THERAPY.” From the resulting set of files, the section on therapeutic procedures was extracted using a regular expression, if such a section existed. This resulted in a total of 23,724 notes, some of which were empty or lacked pertinent information.

The method of section extraction has a few minor limitations. Because the regular expression used to locate these sections assumes a structure in the notes that is not always present, it is possible that a file may contain additional text from other sections of the original note in rare instances. All sections used in the creation of the gold-standard labels were manually examined to ensure the absence of these errors. It is also possible that some therapeutic procedures’ sections are completely omitted from the note due to copy-and-paste errors made by their authors.

Because many of the extracted sections were very brief or lacked relevant information, we developed a method to create a more robust set of sections by extracting keywords. Initially, concepts were organized into 9 categories based on the clinical ontology. Each category was then assigned a list of keywords. A section was considered to mention a category if it contained at least 1 of the keywords. Consequently, each section was assigned a score between 0 and 9 based on the number of categories mentioned. All sections with a score of 9 and a random selection of notes with a score of 8 were extracted to generate 300 enriched sections that were anticipated to be relatively dense in information. In addition, 300 random sections were selected, excluding those with a length of fewer than 200 characters in order to reduce the likelihood of omissions.

### Gold-Standard Data Set Creation

Gold standard labels were developed by 2 clinical experts in the field of physical therapy (PD and BA) under the supervision of a senior clinical expert in physical therapy (AB). Each annotator was given a set of guidelines on how to label sections and was told to refer to the clinical ontology for examples of each concept to label. Instructions were given to label explicit mentions of each concept, and inferences were only to be made when specified. For example, the concepts under the categories exercise type and positioning were each given several common keywords that indicate exercises that relate to them. The annotators were given identical batches of 20 randomly selected sections to annotate, and the interannotator agreement was calculated using Fleiss κ. This process was repeated for a total of 3 batches, after which all 3 annotators achieved an interannotator agreement greater than 0.7. Throughout this process, the annotation guidelines were revised, and the structure of the labels was finalized. Once sufficient agreement was reached, 50 sections from the enriched set and 50 more from the random set were given to each annotator, totaling 300 distinct annotated sections. These sections were then split randomly into a training set consisting of 125 sections from each of the original sets and a test set consisting of the remaining 50 sections. The details of this corpus are included in Textbox 1, which outlines the total word count, the number of distinct words, and 2 examples of the data.

**Summary of the annotated corpus.**

Total words: 74,104

Total distinct words: 2371

**Deidentified note example 1:**

- “1: AROM right elbow flx/ext HEP (right arm supported on table) 2: AROM right wrist flx/ext HEP 3: AROM right forearm pronation/supination HEP 4: Thumb opposition HEP 5: Seated AAROM table slide??”

**Deidentified note example 2:**

- “1: foam balance (heel/toe rocking): x 30 2: step taps with 2 taps from foam 12“”“” block: x 20 B/L 3: tandem walking: 25' x 2 4: backward walking: 25' x 2 5: foam Lunges: x 20 B/L 6: Dips 4“”“” stair: 2x10 B/L 7: side stepping green TB 10 ft x5 each direction 9: bridging with LLE leg lift 1“”“” off mat x10 10: tandem stance on foam x 1' 11: Nustep: L5 x 10' (LEs only)”

### Rule-Based NLP

The first NLP method we developed was a named entity recognition (NER) algorithm using MedTagger (OHNLP), which is a software that uses rule-based methods to segment documents and extract named entity information with regular expressions [14]. We used this tool to detect the categories outlined in the ontology by creating explainable rules to extract the physical rehabilitation exercise information and compare it against the gold-standard labels. For each rule defined in the algorithm, MedTagger identified spans of text that matched the expression as well as the corresponding category and concept predicted for that text. We initiated the rules using simple keywords in the clinical ontology as defined in Table 2 and then refined the rules using the training set of the gold-standard notes.

### Machine Learning–Based NLP

In addition to attempting to automate the annotation of clinical notes with exercise information, several sequence-level binary classification methods were explored to predict whether a specific concept is mentioned in a given span of text at least once according to the gold-standard labels. Here, a sequence is defined as a string of text within a section that describes an individual exercise. As the therapeutic procedures are documented as numbered lists, it is assumed that each enumerated item that contains text constitutes a single procedure for the purpose of this study. The aim was to extract these procedures from sections and then classify each according to which concepts they mention.

For this task, the sequences provided in the gold-standard data were used as raw input, and targets were defined using the labels that were associated with each sequence. These labels consisted of 101 concepts as given by the clinical ontology in Table 2, excluding duration, sets, and reps since these are numeric types unfit for binary classification tasks. Because the postprocessed output from MedTagger was formatted in a similar manner to the gold-standard data for ease of comparison, a similar method was used to create predictions and directly score MedTagger against the true labels for this task. In this manner, we compared our rule-based NLP algorithm against several other methods by redefining the information extraction task as a sequence classification task. The labels of all predicted spans of text were assigned to the section containing it.

A total of 4 machine learning models were trained to perform binary classification on sections, including SVM [15], LR [16], gradient boosting [17], and AdaBoost [18]. We built different machine learning models for different physical rehabilitation exercise concept extraction tasks. This resulted in 101 distinct SVM, LR, gradient boosting, and AdaBoost models each trained to predict a distinct concept. Each model was created using the *scikit-learn* [19] library in Python (version 3; Python Software Foundation). The input for each model was given in a simple uncased bag-of-words vector space fitted to the training set. The LR was performed with a learning rate of 1 × 10^–4^ and balanced class weights. The SVM model used a polynomial kernel with a degree of 2 and also used balanced class weights. The AdaBoost and gradient boosting were performed with the default parameters provided by *scikit-learn*, with 100 and 50 estimators, respectively. All unspecified hyperparameters were kept at the default values used by *scikit-learn*.

### LLM-Based NLP

Recently, LLMs have gained much interest due to their promising results across many NLP tasks and straightforward development pipelines. To measure a baseline for the performance of LLMs on this data set, this study used OpenAI’s ChatGPT, specifically GPT-3.5-turbo [20], which serves as the NLP backend for the first release of ChatGPT. This study accessed ChatGPT through the Microsoft Azure secure cloud service with an approved institutional review board.

For each of the concepts to be identified, a separate prompt was created asking the model to identify whether or not that concept is mentioned in the given sections. We note that the sections used in this exercise were fully deidentified and contained no sensitive information. One of the primary advantages of using LLMs to perform NLP tasks is that they can yield results comparable to other methods with much less time spent on fine-tuning and other manual design tasks. Therefore, in order to evaluate the practicality of using this method in an application with constraints on time or available machine learning expertise, prompts were automatically generated using a template and the name assigned to each label. Two simple prompting techniques were compared: a few-shot prompting technique that included example dialogue for 2 positive examples and 2 negative examples [21] and a zero-shot prompt that omitted any examples [22]. Prompts were created by providing a list of dialogue strings that can be attributed to the user providing the prompt, the model giving the response, and a third party giving instructions to the model, hereafter named System. The following is an example of a few-shot prompt that was automatically generated and sent to ChatGPT.

System: You are an assistant assigned to determine if a given text segment from a medical record contains mentions of Active Range of Motion. You must answer yes or no.

User: 7: [PERSONALNAME] maze - AROM LUE - 3 rep

*Model: Yes.*

User: 23: / Supine on mat / dowel rod no wt above head for triceps

*Model: No.*

User: 2: AROM Right wrist ext/flex - 20 x

*Model: Yes.*

User: 24: SL HS activation- with tapping and suspended to limit friction x10 DEFERRED

*Model: No.*

User: 14: SLR- 2x10 deferred to HEP

Prompts were made in this manner using 4 dialogue exchanges, switching out the last line for the section to be classified. Any concepts that were labeled fewer than 2 times were excluded from the experiment because not enough examples could be generated for the few-shot prompt. A total of 3 additional concepts were excluded relating to whether a procedure was performed in-office, at home, or not at all, as well as the 3 numeric concepts. This left a total of 82 concepts to be extracted.

## Results

Because the clinical ontology covered a broad range of concepts within this domain, many of these concepts were very sparse in our data. More than half of the 101 concepts were present in fewer than 10 exercise descriptions in the train or test sets; these concepts have been omitted from the results. Table 3 contains a breakdown of the *F*_1_-scores for each machine learning method, as well as the performance of the rule-based NLP algorithm on the NER task, for each of the remaining 40 concepts. See Multimedia Appendix 1 for the results on all concepts. The best-performing machine learning model is shown in bold for each concept.

**Table 3:** Binary *F*_1_-scores of each algorithm on the test set (50 documents).

Category and concept			RBNLP^a^ NER^b^, n		RBNLP sequence, n	LR^c^, n		SVM^d^, n		AdaBoost, n		Gradient boosting, n		ChatGPT (few-shot), n		ChatGPT (zero-shot), n		Training set size, n		Test set size, n
**Description**																				
	Performed in-office	0.957		0.976		0.970	0.960		0.977		0.983^e^		N/A^f^		N/A		2464		497	
	Home exercise program	0.986^e^		0.986^e^		0.986^e^	0.938		0.986^e^		0.986^e^		N/A		N/A		93		34	
	Not performed	0.949		0.949		0.923	0.909		0.936		0.950^e^		N/A		N/A		1295		206	
**ROM^g^**																				
	Active	0.839		0.830		0.824	0.840		0.863^e^		0.863^e^		0.321		0.109		103		22	
	Active-assisted	0.769		0.769		0.800	0.791		0.837		0.857^e^		0.543		0.210		160		24	
	Passive	0.952		0.938		0.970^e^	0.903		0.938		0.970^e^		0.552		0.198		121		16	
**Side**																				
	Right side	0.912		0.975^e^		0.674	0.851		0.628		0.680		0.912		0.878		548		97	
	Left side	0.912		0.937^e^		0.763	0.823		0.721		0.752		0.823		0.832		462		134	
	Bilateral	0.772		0.907^e^		0.559	0.474		0.667		0.659		0.706		0.723		260		51	
**Location**																				
	Upper extremity	0.847		0.939^e^		0.879	0.847		0.901		0.876		0.291		0.241		285		47	
	Lower extremity	0.955		0.936		0.936	0.930		0.966		0.978^e^		0.378		0.339		223		44	
	Hip	0.949		0.947		0.973^e^	0.973^e^		0.943		0.972		0.403		0.806		168		36	
	Knee	0.950		0.950		0.919	0.882		0.974^e^		0.974^e^		0.469		0.434		108		19	
	Ankle	1.000^e^		1.000^e^		0.923	0.600		1.000^e^		1.000^e^		0.607		0.262		55		14	
	Shoulder	0.936		0.977^e^		0.952	0.952		0.953		0.953		0.744		0.548		224		44	
	Scapula	0.833^e^		0.833^e^		0.783	0.700		0.833^e^		0.833^e^		0.525		0.607		72		10	
	Elbow	0.967^e^		0.963		0.963	0.943		0.923		0.923		0.848		0.447		147		26	
	Forearm	0.815		0.833		0.870	0.952^e^		0.870		0.952^e^		0.151		0.204		86		10	
	Wrist	0.902^e^		0.898		0.826	0.773		0.875		0.875		0.600		0.314		129		23	
	Hand	0.951^e^		0.944		0.926	0.848		0.925		0.949		0.438		0.574		243		68	
**Plane**																				
	Abduction	0.976		0.985^e^		0.971	0.937		0.971		0.971		0.576		0.839		170		33	
	Anterior	0.545		0.545		0.750^e^	0.667		0.750^e^		0.667		0.221		0.195		22		10	
	Backward	0.727		0.720		0.688	0.800		0.952^e^		0.846		0.720		0.790		92		11	
	Extension	0.980		0.980		0.979	0.933		0.989^e^		0.989^e^		0.556		0.684		266		48	
	External rotation	0.897		0.917^e^		0.870	0.818		0.870		0.870		0.655		0.543		74		11	
	Flexion	0.956		0.947		0.964^e^	0.955		0.964^e^		0.964^e^		0.757		0.615		327		55	
	Forward	0.977^e^		0.974		0.857	0.865		0.950		0.900		0.667		0.729		148		19	
	Lateral	0.577		0.588		0.786	0.837		0.870^e^		0.851		0.546		0.373		132		23	
	Supination	0.923^e^		0.917		0.880	0.917		0.917		0.917		0.550		0.480		82		11	
**Exercise type**																				
	Upper extremity strength	0.913^e^		0.913^e^		0.840	0.791		0.913^e^		0.894		0.272		0.166		138		21	
	Lower extremity strength	0.926		0.969^e^		0.913	0.894		0.924		0.894		0.449		0.332		447		97	
	Trunk or core strength	0.897		0.889^e^		0.692	0.471		0.471		0.700		0.104		0.090		35		12	
	Range of motion	0.853		0.876^e^		0.842	0.843		0.725		0.674		0.301		0.153		257		53	
	Flexibility or mobility	0.962		0.974^e^		0.909	0.857		0.947		0.949		0.279		0.147		178		38	
	Balance or vestibular	0.787		0.752		0.852	0.809		0.882		0.939^e^		0.597		0.470		351		47	
	Gait training	0.808		0.837		0.837	0.814		0.851		0.860^e^		0.626		0.529		310		47	
	Functional mobility	0.775		0.831^e^		0.727	0.750		0.691		0.780		0.220		0.182		204		33	
**Purpose**																				
	Simulated	0.769		0.769		0.870^e^	0.762		0.857		0.870^e^		0.688		0.667		48		10	
**Positioning**																				
	Weight bearing	0.788		0.833		0.876^e^	0.867		0.857		0.871		0.197		0.282		255		43	
	Non-weight bearing	0.931		0.932		0.916	0.918		0.946^e^		0.923		0.283		0.038		539		91	
Average			0.878		0.891^e^	0.861		0.835		0.875		0.883		0.502		0.433		283		53

^a^RBNLP: rule-based natural language processing.

^b^NER: named entity recognition.

^c^LR: logistic regression.

^d^SVM: support vector machine.

^e^The best performance for each entity.

^f^N/A: not applicable.

^g^ROM: range of motion.

The rule-based NLP’s performance on the sequence classification task was similar to its performance on the NER task. Instances of higher performance in sequence classification compared to NER can be partially explained by mismatches in predicted spans and their labels affecting NER accuracy, yet still allowing for correct overall text section classification. The rule-based algorithm tied with or outperformed the other models on half of the concepts in Table 3. Among the machine learning models, gradient boosting performed nearly as well, achieving the highest *F*_1_-score on 18 concepts.

In addition to these concepts, the rule-based NLP algorithm also predicted the spans of durations, sets, and reps. Since these categories do not have any specific concepts assigned to them, the number presented in each span was used instead as a comparison against the true label, converting minutes to seconds where applicable. This resulted in *F*_1_-scores of 0.65, 0.58, and 0.88, respectively. It is important to note that we limited the experiments for “Duration,” “Sets,” and “Reps” exclusively to rule-based algorithms because these categories inherently involve numeric data, which align well with the deterministic and pattern-based nature of rule-based approaches.

Gradient boosting demonstrated the best performance for identifying range of motion (ROM) concepts and determining the location of exercise (performed in-office, home exercise program, and not performed) with *F*_1_-scores of 0.863 for active ROM; 0.857 for active-assisted ROM; and 0.977, 0.986, and 0.950, respectively, for the locations. The rule-based natural language processing algorithm outperformed machine learning models in detecting sides of the body with *F*_1_-scores of 0.975 for the right side and 0.937 for the left side, and it also performed the best on most exercise types, except for balance or vestibular and gait training concepts, which were classified best by gradient boosting with *F*_1_-scores of 0.939 and 0.860, respectively. The LR obtained a strictly higher score than other methods in the weight-bearing exercise concept with an *F*_1_-score of 0.876. The AdaBoost got a strictly higher score on 3 concepts, notably on non–weight bearing positioning with an *F*_1_-score of 0.946. The SVM model did not score higher than other models but had 3 ties, indicating competitive performance.

These findings indicate that the rule-based approach is particularly effective for certain types of exercises, with superior performance in most categories. However, gradient boosting demonstrated strength in more complex categorizations such as balance or vestibular and gait training, where understanding nuanced differences is crucial.

For the LLM-based NLP, the results show that both zero-shot prompts and few-shot prompts result in high recall scores that sometimes exceed other methods. However, precision was quite low for most concepts, and *F*_1_-scores did not exceed every other method for any concept. However, ChatGPT did occasionally outperform some of the simpler machine learning models and, on 1 occasion, even outperformed the rule-based algorithm (on the backward plane of motion concept). The average precision over all 82 concepts tested was 0.33 for the zero-shot approach and 0.27 for the few-shot approach. The average recall was 0.8 for the zero-shot approach and 0.82 for the few-shot approach. This resulted in average *F*_1_-scores of 0.37 and 0.35, respectively, indicating that the zero-shot approach was slightly better on average than the few-shot approach. However, the few-shot approach performed the best for all but 10 concepts. The reason the zero-shot method performed better on average is thus due to the fact that it shows significant improvement on a few specific concepts, such as hip, scapula, hand, abduction, and extension.

## Discussion

### Observations

As indicated by the high performance of the machine learning models on many of the concepts, the task of extracting information from exercise descriptions was not complex. Although some of these concepts could be extracted effectively using straightforward rules or a small machine learning model, there were also many cases where clinical notes appeared ambiguous without context. For instance, the abbreviation “SL” could be interpreted as “single leg” or “side-lying” depending on the exercise being described. In addition, “L” could mean “left” or “lateral,” which explains why the rule-based NLP algorithm performed slightly worse when classifying left versus right. The use of single letters as abbreviations, especially “A” as a shorthand for “anterior,” could cause issues in machine learning algorithms without careful consideration. It would be possible to increase the performance of the rule-based algorithm by further tuning the rules to search for context clues at other points in the document, but this could potentially cause the rules to overfit the training set. Of particular interest are the numeric data present in duration, sets, and reps. These are particularly tricky to extract since they are expressed in a wide variety of ways by different physicians. It can be difficult to define what sets and reps are depending on the exercise, and sometimes one or both are not well-defined at all. Additionally, the use of apostrophes and quotes can either indicate measurements of time or distance, once again requiring context to disambiguate. Mentions of distance were not annotated in the gold-standard labels, but it is important in measuring the intensity of some exercises, so we plan to include it in the future.

Some of the misclassifications of the rule-based algorithm are due to inaccuracies in the gold-standard data set. For instance, many false positives produced by the rule-based algorithm appeared to be concepts that were missed by the annotators. There were also a few minor errors that could be explained by a mouse slip, including a span of text being assigned the wrong concept or a span excluding the last letter in a word. There were also some spelling mistakes in the notes themselves; common instances were explicitly mentioned in the rules to increase precision. Preprocessing clinical notes to correct spelling mistakes might be useful to improve results, although this creates a risk of incorrect changes being made to uncommon words. All of these errors were not particularly common throughout the labels, but they could have a significant effect on concepts that are already uncommon in the data.

Another obstacle that obscured some of the signals in the data came from the deidentification process. In addition to removing names, addresses, and other protected information from these documents, many other tokens and phrases were mistakenly removed, including equipment names and numbers denoting indices in a list. These were replaced with placeholder tokens such as “[ADDRESS]” or “[PERSONALNAME].” The low precision of the deidentification process caused some relevant information to be obfuscated or entirely erased from notes.

During the data annotation, we found that many of the concepts identified as relevant in this domain were not well documented in the data we extracted for annotation. This could be due in part to the fact that the data were only collected from patients who had a stroke, but this is not expected to be the main reason because patients who had a stroke can have a wide variety of musculoskeletal problems, resulting in a correspondingly wide variety of treatments being mentioned in clinical notes [23]. The other reason the data set lacks many of these concepts could be that they are rarely mentioned in these particular sections of clinical notes, either because they are not common enough to appear in many records at all or because they are mentioned more often in other sections. Thus, future research could focus on improving extraction methods to focus more on these uncommon concepts or include information from outside of the exercise descriptions.

In addition to ChatGPT for the LLM-based NLP approach, we also fine-tuned a Bidirectional Encoder Representations from Transformers (BERT) model with the task of categorizing the physical rehabilitation exercise concept. The BioClinicalBERT model [24] was used, which was pretrained on Medical Information Mart for Intensive Care-III (MIMIC-III) [25]. However, the amount of data collected seemed insufficient to make the model perform comparably to simpler methods. The model with the highest *F*_1_-score on the validation set had an average *F*_1_-score of 0.05 across all concepts on the test set. It accurately predicted in-office exercise performance with an *F*_1_-score of 0.72. However, the performance on the remaining 100 concepts ranged only from 0 to 0.35. Therefore, we did not include this approach in the experimental comparison.

### Limitations and Future Work

One limitation in this research was the necessary exclusion of “Duration,” “Number of Sets,” and “Number of Reps” from our machine learning–based NLP analysis due to their numeric nature, rendering them unsuitable for binary classification tasks. In future work, we plan to incorporate regression models or specialized classification techniques capable of handling numeric data. We also plan to expand our research to include additional variables such as stroke duration and severity, recognizing their potential to significantly enhance the prediction accuracy and effectiveness of rehabilitation strategies.

Furthermore, another limitation of this study is that we did not consider technique names and their association with specific motion types in rehabilitation exercise notes. For instance, we encountered the text “1: Standing AAROM PNF exercise D1/D2 flexion - 20 x” during annotation but did not annotate the technique name PNF (proprioceptive neuromuscular facilitation). To address this, in future work, we intend to develop a supplementary module for our algorithm that can effectively extract and map popular technique names to their corresponding motion types and categories, thereby enhancing the comprehensiveness and applicability of the algorithm.

Moreover, we plan to implement a robust standardized extraction protocol in the next version of our algorithm to mitigate the omission of therapeutic procedure sections due to copy-and-paste errors. This protocol will include multiple checks for consistency and completeness and will be assessed through a pilot study to ensure its reliability and accuracy. To enhance our model’s generalizability amid varied note-writing practices across rehabilitation facilities, future research will also focus on diversifying data sources, refining adaptability to diverse writing styles and terminologies, and conducting extensive validation studies in a range of settings to improve performance. Through continuous monitoring and refinement of our extraction process, we are committed to enhancing the reliability and validity of our data, thereby strengthening the overall quality and impact of our research.

### Conclusions

In this study, we developed and evaluated several NLP algorithms to extract physical rehabilitation exercise information from clinical notes of patients who had stroke. We first created a novel and comprehensive clinical ontology to represent physical rehabilitation exercise in clinical notes and then developed a variety of NLP algorithms leveraging state-of-the-art techniques, including rule-based NLP algorithms, machine learning–based NLP algorithms, and LLM-based NLP algorithms. The experiments on the clinical notes of a cohort of patients who had a stroke showed that the rule-based NLP algorithm had the best performance for most of the physical rehabilitation exercise concepts. Among all machine learning models, gradient boosting achieved the best performance on a majority of concepts. On the other hand, the rule-based NLP performed well for extracting handled durations, sets, and reps, while gradient boosting excelled in ROM and location detection. The LLM-based NLP achieved high recall with zero-shot and few-shot prompts but low precision and *F*_1_-scores. It occasionally outperformed simpler models and once bet the rule-based algorithm.

## Appendix

Multimedia Appendix 1 Full binary *F*_1_-scores of each algorithm on the test set (50 documents) and additional results from the ChatGPT experiment.

## Abbreviations

BERT: Bidirectional Encoder Representations from Transformers
ICF: International Classification of Functioning, Disability, and Health
LLM: large language model
LR: logistic regression
MIMIC-III: Medical Information Mart for Intensive Care-III
NER: named entity recognition
NLP: natural language processing
PNF: proprioceptive neuromuscular facilitation
ROM: range of motion
SNOMED CT: Systematized Nomenclature of Medicine—Clinical Terms
SVM: support vector machine
UMLS: Unified Medical Language System
UPMC: University of Pittsburgh Medical Center
