# Supplementary material for: Mining Clinical Notes for Physical Rehabilitation Exercise Information: Natural Language Processing Algorithm Development and Validation Study
Source: JMIR Med Inform. 2024 Apr 3;12:e52289. doi: 10.2196/52289 (PMC11024747; doi:10.2196/52289)
Supplement: Multimedia Appendix 1 [file medinform_v12i1e52289_app1.docx]

(RBNLP: Rule-based NLP, LR: Linear Regression, SVM: Support Vector Machine, AB: AdaBoost, GB: Gradient Boosting, NER: Named Entity Recognition)

**Table S1.** Full binary *F*_1_-scores of each algorithm on the test set (50 documents).

| Category | Concept | RBNLP NER | RBNLP  Sequence | LR | SVM | AB | Gradient  Boosting | ChatGPT  (few-shot) | ChatGPT  (zero-shot) | Training  Set Size | Test Set Size |
| --- | --- | --- | --- | --- | --- | --- | --- | --- | --- | --- | --- |
| Description | Performed In-Office | 0.957 | 0.976 | 0.97 | 0.96 | 0.977 | 0.983 | N/A | N/A | 2464 | 497 |
|  | Home Exercise  Program | 0.986 | 0.986 | 0.986 | 0.938 | 0.986 | 0.986 | N/A | N/A | 93 | 34 |
|  | Not Performed | 0.949 | 0.949 | 0.923 | 0.909 | 0.936 | 0.95 | N/A | N/A | 1295 | 206 |
| ROM | Range of Motion | 0.923 | 0.889 | 0.667 | 0.667 | 1 | 1 | 0.071 | 0.022 | 25 | 5 |
|  | Active | 0.839 | 0.83 | 0.824 | 0.84 | 0.863 | 0.863 | 0.321 | 0.109 | 103 | 22 |
|  | Active-Assisted | 0.769 | 0.769 | 0.8 | 0.791 | 0.837 | 0.857 | 0.543 | 0.21 | 160 | 24 |
|  | Passive | 0.952 | 0.938 | 0.97 | 0.903 | 0.938 | 0.97 | 0.552 | 0.198 | 121 | 16 |
| Side of Body | Right | 0.912 | 0.975 | 0.674 | 0.851 | 0.628 | 0.68 | 0.912 | 0.878 | 548 | 97 |
|  | Left | 0.912 | 0.937 | 0.763 | 0.823 | 0.721 | 0.752 | 0.823 | 0.832 | 462 | 134 |
|  | Bilateral | 0.772 | 0.907 | 0.559 | 0.474 | 0.667 | 0.659 | 0.706 | 0.723 | 260 | 51 |
|  | Unilateral | 0.75 | 0.75 | 0.25 | 0.4 | 0 | 0 | 0.095 | 0.069 | 26 | 4 |
|  | Contralateral | 0 | 0 | 0 | 0 | 0 | 0 | N/A | N/A | 1 | 0 |
|  | Ipsilateral | 0 | 0 | 0 | 0 | 0 | 0 | N/A | N/A | 0 | 0 |
| Location | Arm | 0.847 | 0.939 | 0.879 | 0.847 | 0.901 | 0.876 | 0.291 | 0.241 | 285 | 47 |
|  | Leg | 0.955 | 0.936 | 0.936 | 0.93 | 0.966 | 0.978 | 0.378 | 0.339 | 223 | 44 |
|  | Hip | 0.949 | 0.947 | 0.973 | 0.973 | 0.943 | 0.972 | 0.403 | 0.806 | 168 | 36 |
|  | Thigh | 1 | 1 | 1 | 1 | 1 | 1 | 0.038 | 0.019 | 7 | 3 |
|  | Knee | 0.95 | 0.95 | 0.919 | 0.882 | 0.974 | 0.974 | 0.469 | 0.434 | 108 | 19 |
|  | Ankle | 1 | 1 | 0.923 | 0.6 | 1 | 1 | 0.607 | 0.262 | 55 | 14 |
|  | Foot | 0.923 | 0.909 | 0.667 | 0.5 | 0.667 | 0.667 | 0.099 | 0.065 | 26 | 6 |
|  | Heel | 0.857 | 0.857 | 0.857 | 0.947 | 0.857 | 0.9 | 0.634 | 0.321 | 62 | 9 |
|  | Toe | 0.727 | 0.727 | 0.615 | 0.857 | 0.889 | 1 | 0.388 | 0.551 | 53 | 4 |
|  | Shoulder | 0.936 | 0.977 | 0.952 | 0.952 | 0.953 | 0.953 | 0.744 | 0.548 | 224 | 44 |
|  | Scapula | 0.833 | 0.833 | 0.783 | 0.7 | 0.833 | 0.833 | 0.525 | 0.607 | 72 | 10 |
|  | Elbow | 0.967 | 0.963 | 0.963 | 0.943 | 0.923 | 0.923 | 0.848 | 0.447 | 147 | 26 |
|  | Forearm | 0.815 | 0.833 | 0.87 | 0.952 | 0.87 | 0.952 | 0.151 | 0.204 | 86 | 10 |
|  | Wrist | 0.902 | 0.898 | 0.826 | 0.773 | 0.875 | 0.875 | 0.6 | 0.314 | 129 | 23 |
|  | Hand | 0.951 | 0.944 | 0.926 | 0.848 | 0.925 | 0.949 | 0.438 | 0.574 | 243 | 68 |
|  | Thumb | 0.933 | 0.933 | 0.933 | 0.923 | 0.933 | 0.933 | 0.376 | 0.304 | 30 | 7 |
|  | Head | 1 | 1 | 0.5 | 0.286 | 0.5 | 0.727 | 0.427 | 0.41 | 16 | 6 |
|  | Neck | 1 | 1 | 0 | 0 | 0.909 | 0.769 | 0.129 | 0.265 | 10 | 6 |
|  | Chest | 1 | 1 | 0.667 | 0.667 | 0.857 | 0.4 | 0.129 | 0.353 | 9 | 4 |
|  | Abdomen | 0 | 0 | 0 | 0 | 0 | 0 | 0.042 | 0.099 | 4 | 0 |
|  | Lower Back | 1 | 1 | 0 | 0 | 1 | 1 | 0.073 | 0.122 | 8 | 1 |
| Plane of  Motion | Abduction | 0.976 | 0.985 | 0.971 | 0.937 | 0.971 | 0.971 | 0.576 | 0.839 | 170 | 33 |
|  | Adduction | 0.857 | 0.857 | 0.8 | 0.706 | 0.9 | 0.857 | 0.23 | 0.267 | 34 | 9 |
|  | All planes | 0 | 0 | 0 | 0 | 0 | 0 | 0.014 | 0.19 | 4 | 0 |
|  | Anterior | 0.545 | 0.545 | 0.75 | 0.667 | 0.75 | 0.667 | 0.221 | 0.195 | 22 | 10 |
|  | Backward | 0.727 | 0.72 | 0.688 | 0.8 | 0.952 | 0.846 | 0.72 | 0.79 | 92 | 11 |
|  | Clockwise | 1 | 1 | 0.8 | 0 | 1 | 1 | 0.619 | 0.884 | 13 | 3 |
|  | Closure | 0 | 0 | 0 | 0 | 0 | 0 | N/A | N/A | 0 | 0 |
|  | Counterclockwise | 1 | 1 | 0.8 | 0 | 1 | 1 | 0.622 | 0.364 | 14 | 3 |
|  | Depression | 0 | 0 | 0 | 0 | 0 | 0 | N/A | N/A | 1 | 0 |
|  | Diagonal | 0 | 1 | 0 | 0 | 0 | 0 | 0 | 1 | 1 | 1 |
|  | Distraction | 0 | 0 | 0 | 0 | 0 | 0 | N/A | N/A | 0 | 0 |
|  | Dorsiflexion | 0.923 | 0.923 | 0.923 | 0.833 | 0.923 | 0.923 | 0.356 | 0.189 | 34 | 6 |
|  | Downward Rotation | 0 | 0 | 0 | 0 | 0 | 0 | N/A | N/A | 0 | 0 |
|  | Elevation | 1 | 1 | 0 | 0 | 1 | 1 | 0.159 | 0.346 | 8 | 1 |
|  | Eversion | 1 | 1 | 0.8 | 0.889 | 0.889 | 1 | 0.897 | 0.821 | 13 | 5 |
|  | Extension | 0.98 | 0.98 | 0.979 | 0.933 | 0.989 | 0.989 | 0.556 | 0.684 | 266 | 48 |
|  | External Rotation | 0.897 | 0.917 | 0.87 | 0.818 | 0.87 | 0.87 | 0.655 | 0.543 | 74 | 11 |
|  | Flexion | 0.956 | 0.947 | 0.964 | 0.955 | 0.964 | 0.964 | 0.757 | 0.615 | 327 | 55 |
|  | Forward | 0.977 | 0.974 | 0.857 | 0.865 | 0.95 | 0.9 | 0.667 | 0.729 | 148 | 19 |
|  | Gravity elimination | 0 | 0 | 0 | 0 | 0 | 0 |  |  | 0 | 0 |
|  | Horizontal | 0.75 | 1 | 0.2 | 0 | 0.333 | 0.25 | 0.236 | 0.493 | 43 | 4 |
|  | Horizontal  Abduction | 0 | 0 | 0 | 0 | 0 | 0 | N/A | N/A | 1 | 0 |
|  | Horizontal  Adduction | 0 | 0 | 0 | 0 | 0 | 0 | N/A | N/A | 0 | 0 |
|  | Internal Rotation | 0.857 | 0.833 | 0.833 | 0.727 | 0.833 | 0.833 | 0.373 | 0.449 | 41 | 5 |
|  | Inversion | 1 | 1 | 0.8 | 0.75 | 0.889 | 0.889 | 0.489 | 0.811 | 10 | 5 |
|  | Lateral | 0.577 | 0.588 | 0.786 | 0.837 | 0.87 | 0.851 | 0.546 | 0.373 | 132 | 23 |
|  | Lateral Flexion | 1 | 1 | 0 | 0 | 1 | 1 | 0.013 | 0.023 | 6 | 1 |
|  | Medial | 0.75 | 0.75 | 0.8 | 0.571 | 0.727 | 0.667 | 0.081 | 0.16 | 26 | 5 |
|  | Opposition | 0.75 | 0.75 | 0.889 | 0.889 | 0.75 | 0.75 | 0.074 | 0.667 | 15 | 4 |
|  | Plantarflexion | 1 | 1 | 1 | 0.857 | 1 | 1 | 0.073 | 0.124 | 11 | 4 |
|  | Posterior | 0.941 | 0.941 | 0.615 | 0.5 | 0.875 | 0.824 | 0.178 | 0.333 | 22 | 9 |
|  | Pronation | 0.444 | 0.444 | 0.923 | 0.833 | 0.923 | 0.857 | 0.45 | 0.407 | 30 | 6 |
|  | Protraction | 1 | 1 | 0.667 | 0.857 | 1 | 0.857 | 0.423 | 0.889 | 15 | 3 |
|  | Radial Deviation | 0.667 | 0.667 | 0 | 0.667 | 0.667 | 0.5 | 0.077 | 0.045 | 3 | 1 |
|  | Retraction | 0.875 | 0.875 | 0.875 | 0.933 | 0.636 | 0.875 | 0.516 | 0.963 | 39 | 7 |
|  | Rotation | 0.769 | 0.769 | 0.444 | 0.286 | 0.4 | 0.833 | 0.107 | 0.138 | 16 | 6 |
|  | Scaption | 0.667 | 0.667 | 0.667 | 0 | 0.667 | 0.5 | 0.485 | 0.237 | 17 | 1 |
|  | Supination | 0.923 | 0.917 | 0.88 | 0.917 | 0.917 | 0.917 | 0.55 | 0.48 | 82 | 11 |
|  | Ulnar Deviation | 0.667 | 0.667 | 0.091 | 0.667 | 0 | 0 | 0 | 0.058 | 1 | 1 |
|  | Upward Rotation | 0 | 0 | 0 | 0 | 0 | 0 | N/A | N/A | 0 | 0 |
|  | Vertical | 1 | 1 | 0.222 | 0 | 0.5 | 0.333 | 0.346 | 0.759 | 52 | 2 |
| Exercise Type | Upper Extremity  Strength | 0.913 | 0.913 | 0.84 | 0.791 | 0.913 | 0.894 | 0.272 | 0.166 | 138 | 21 |
|  | Lower Extremity  Strength | 0.926 | 0.969 | 0.913 | 0.894 | 0.924 | 0.894 | 0.449 | 0.332 | 447 | 97 |
|  | Trunk/Core Strength | 0.897 | 0.889 | 0.692 | 0.471 | 0.471 | 0.7 | 0.104 | 0.09 | 35 | 12 |
|  | Scapular Strength | 0.471 | 0.706 | 0.778 | 0.462 | 0.462 | 0.462 | 0.202 | 0.137 | 43 | 9 |
|  | Range of Motion | 0.853 | 0.876 | 0.842 | 0.843 | 0.725 | 0.674 | 0.301 | 0.153 | 257 | 53 |
|  | Flexibility/Mobility | 0.962 | 0.974 | 0.909 | 0.857 | 0.947 | 0.949 | 0.279 | 0.147 | 178 | 38 |
|  | Balance/Vestibular | 0.787 | 0.752 | 0.852 | 0.809 | 0.882 | 0.939 | 0.597 | 0.47 | 351 | 47 |
|  | Gait Training | 0.808 | 0.837 | 0.837 | 0.814 | 0.851 | 0.86 | 0.626 | 0.529 | 310 | 47 |
|  | Cardio/Aerobic | 1 | 1 | 0.706 | 0.714 | 0.824 | 0.778 | 0.691 | 0.556 | 55 | 9 |
|  | Functional Mobility | 0.775 | 0.831 | 0.727 | 0.75 | 0.691 | 0.78 | 0.22 | 0.182 | 204 | 33 |
| Exercise  Purpose | Strength | 0.526 | 0.526 | 0.615 | 0.6 | 0.333 | 0.25 | 0.042 | 0.029 | 29 | 5 |
|  | Power | 0 | 0 | 0 | 0 | 0 | 0 | N/A | N/A | 0 | 0 |
|  | Endurance | 0 | 0 | 0 | 0 | 0 | 0 | N/A | N/A | 1 | 0 |
|  | Joint Mobility | 0.667 | 0.667 | 1 | 1 | 1 | 0.667 | 0.02 | 0.009 | 5 | 1 |
|  | Joint Alignment | 0 | 0 | 0 | 0 | 0 | 0 | N/A | N/A | 0 | 0 |
|  | Muscle Flexibility | 0 | 0 | 0 | 0 | 0 | 0 | N/A | N/A | 0 | 0 |
|  | Cardio | 0 | 0 | 0 | 0 | 0 | 0 | 0 | 0 | 2 | 0 |
|  | Pulmonary | 0 | 0 | 0 | 0 | 0 | 0 | N/A | N/A | 0 | 0 |
|  | Agility | 0 | 0 | 0 | 0 | 0 | 0 | 0.143 | 0.563 | 6 | 0 |
|  | Vestibular | 0 | 0 | 0 | 0 | 0 | 0 | N/A | N/A | 0 | 0 |
|  | Fine Motor | 0.667 | 0.667 | 0.667 | 0.667 | 0.667 | 0.8 | 0.043 | 0.03 | 18 | 2 |
|  | Motor Control | 0 | 0 | 0 | 0 | 0 | 0 | 0.003 | 0.003 | 3 | 0 |
|  | Perception | 0 | 0 | 0 | 0 | 0 | 0 | 0 | 0.115 | 2 | 0 |
|  | Simulated | 0.769 | 0.769 | 0.87 | 0.762 | 0.857 | 0.87 | 0.688 | 0.667 | 48 | 10 |
| Positioning | Weight Bearing | 0.788 | 0.833 | 0.876 | 0.867 | 0.857 | 0.871 | 0.197 | 0.282 | 255 | 43 |
|  | Non-Weight Bearing | 0.931 | 0.932 | 0.916 | 0.918 | 0.946 | 0.923 | 0.283 | 0.038 | 539 | 91 |
| Average | | 0.668 | 0.687 | 0.569 | 0.535 | 0.632 | 0.628 | 0.354 | 0.374 | 121 | 23 |

**Table S2.** Additional results from the ChatGPT experiment.

|  | | Few-shot | | | | Zero-shot | | | |
| --- | --- | --- | --- | --- | --- | --- | --- | --- | --- |
| Category | Concept | Accuracy | Precision | Recall | F1 | Accuracy | Precision | Recall | F1 |
| ROM | Range of Motion | 0.836 | 0.037 | 1.000 | 0.071 | 0.441 | 0.011 | 1.000 | 0.022 |
|  | Active Range of Motion | 0.890 | 0.194 | 0.935 | 0.321 | 0.574 | 0.058 | 0.983 | 0.109 |
|  | Active-Assisted Range of Motion | 0.933 | 0.376 | 0.978 | 0.543 | 0.723 | 0.117 | 0.994 | 0.210 |
|  | Passive Range of Motion | 0.951 | 0.383 | 0.985 | 0.552 | 0.805 | 0.110 | 0.982 | 0.198 |
| Side | Right | 0.973 | 0.864 | 0.965 | 0.912 | 0.970 | 0.948 | 0.819 | 0.878 |
|  | Left | 0.956 | 0.869 | 0.781 | 0.823 | 0.956 | 0.825 | 0.839 | 0.832 |
|  | Bilateral | 0.963 | 0.753 | 0.664 | 0.706 | 0.965 | 0.967 | 0.578 | 0.723 |
|  | Unilateral | 0.910 | 0.050 | 0.750 | 0.095 | 0.890 | 0.037 | 0.514 | 0.069 |
| Location | Upper Extremity | 0.706 | 0.177 | 0.820 | 0.291 | 0.670 | 0.146 | 0.693 | 0.241 |
|  | Lower Extremity | 0.842 | 0.244 | 0.832 | 0.378 | 0.767 | 0.206 | 0.957 | 0.339 |
|  | Hip | 0.874 | 0.253 | 0.990 | 0.403 | 0.977 | 0.680 | 0.991 | 0.806 |
|  | Thigh | 0.909 | 0.019 | 1.000 | 0.038 | 0.860 | 0.010 | 1.000 | 0.019 |
|  | Knee | 0.952 | 0.335 | 0.783 | 0.469 | 0.938 | 0.288 | 0.882 | 0.434 |
|  | Ankle | 0.981 | 0.436 | 1.000 | 0.607 | 0.904 | 0.151 | 1.000 | 0.262 |
|  | Foot | 0.897 | 0.052 | 0.893 | 0.099 | 0.827 | 0.034 | 1.000 | 0.065 |
|  | Heel | 0.983 | 0.468 | 0.985 | 0.634 | 0.940 | 0.191 | 1.000 | 0.321 |
|  | Toe | 0.964 | 0.243 | 0.962 | 0.388 | 0.982 | 0.386 | 0.961 | 0.551 |
|  | Shoulder | 0.960 | 0.597 | 0.989 | 0.744 | 0.911 | 0.378 | 0.996 | 0.548 |
|  | Scapula | 0.968 | 0.356 | 1.000 | 0.525 | 0.980 | 0.436 | 1.000 | 0.607 |
|  | Elbow | 0.987 | 0.757 | 0.965 | 0.848 | 0.925 | 0.290 | 0.978 | 0.447 |
|  | Forearm | 0.792 | 0.082 | 0.872 | 0.151 | 0.849 | 0.114 | 0.989 | 0.204 |
|  | Wrist | 0.955 | 0.429 | 0.993 | 0.600 | 0.876 | 0.187 | 0.984 | 0.314 |
|  | Hand | 0.836 | 0.287 | 0.922 | 0.438 | 0.919 | 0.427 | 0.877 | 0.574 |
|  | Thumb | 0.974 | 0.232 | 1.000 | 0.376 | 0.955 | 0.180 | 1.000 | 0.304 |
|  | Head | 0.989 | 0.275 | 0.950 | 0.427 | 0.990 | 0.262 | 0.941 | 0.410 |
|  | Neck | 0.964 | 0.069 | 1.000 | 0.129 | 0.989 | 0.155 | 0.900 | 0.265 |
|  | Chest | 0.970 | 0.069 | 0.909 | 0.129 | 0.995 | 0.261 | 0.545 | 0.353 |
|  | Abdomen | 0.979 | 0.021 | 1.000 | 0.042 | 0.980 | 0.052 | 1.000 | 0.099 |
|  | Lower Back | 0.960 | 0.038 | 1.000 | 0.073 | 0.981 | 0.068 | 0.600 | 0.122 |
| Plane of Motion | Abduction | 0.944 | 0.430 | 0.871 | 0.576 | 0.984 | 0.811 | 0.870 | 0.839 |
|  | Adduction | 0.943 | 0.130 | 1.000 | 0.230 | 0.962 | 0.157 | 0.912 | 0.267 |
|  | All planes | 0.935 | 0.007 | 1.000 | 0.014 | 0.992 | 0.105 | 1.000 | 0.190 |
|  | Anterior | 0.967 | 0.130 | 0.750 | 0.221 | 0.993 | 0.250 | 0.160 | 0.195 |
|  | Backward | 0.988 | 0.753 | 0.691 | 0.720 | 0.990 | 0.922 | 0.692 | 0.790 |
|  | Clockwise | 0.996 | 0.464 | 0.929 | 0.619 | 0.999 | 0.905 | 0.864 | 0.884 |
|  | Counterclockwise | 0.996 | 0.467 | 0.933 | 0.622 | 0.995 | 0.600 | 0.261 | 0.364 |
|  | Diagonal | 0.992 | 0.000 | 0.000 | 0.000 | 1.000 | 1.000 | 1.000 | 1.000 |
|  | Dorsiflexion | 0.975 | 0.225 | 0.861 | 0.356 | 0.923 | 0.104 | 1.000 | 0.189 |
|  | Elevation | 0.983 | 0.086 | 1.000 | 0.159 | 0.988 | 0.209 | 1.000 | 0.346 |
|  | Eversion | 0.999 | 0.813 | 1.000 | 0.897 | 0.998 | 0.696 | 1.000 | 0.821 |
|  | Extension | 0.912 | 0.425 | 0.803 | 0.556 | 0.947 | 0.535 | 0.948 | 0.684 |
|  | External Rotation | 0.983 | 0.514 | 0.901 | 0.655 | 0.968 | 0.374 | 0.988 | 0.543 |
|  | Flexion | 0.955 | 0.683 | 0.849 | 0.757 | 0.910 | 0.453 | 0.955 | 0.615 |
|  | Forward | 0.974 | 0.606 | 0.741 | 0.667 | 0.983 | 0.962 | 0.587 | 0.729 |
|  | Horizontal | 0.939 | 0.135 | 0.955 | 0.236 | 0.992 | 0.750 | 0.367 | 0.493 |
|  | Internal Rotation | 0.968 | 0.232 | 0.955 | 0.373 | 0.977 | 0.302 | 0.875 | 0.449 |
|  | Inversion | 0.995 | 0.324 | 1.000 | 0.489 | 0.998 | 0.682 | 1.000 | 0.811 |
|  | Lateral | 0.966 | 0.487 | 0.622 | 0.546 | 0.905 | 0.242 | 0.808 | 0.373 |
|  | Lateral Flexion | 0.860 | 0.006 | 0.800 | 0.013 | 0.962 | 0.012 | 0.286 | 0.023 |
|  | Medial | 0.949 | 0.045 | 0.370 | 0.081 | 0.995 | 1.000 | 0.087 | 0.160 |
|  | Opposition | 0.916 | 0.039 | 0.882 | 0.074 | 0.998 | 0.769 | 0.588 | 0.667 |
|  | Plantarflexion | 0.931 | 0.038 | 1.000 | 0.073 | 0.952 | 0.066 | 0.938 | 0.124 |
|  | Posterior | 0.992 | 0.211 | 0.154 | 0.178 | 0.995 | 0.600 | 0.231 | 0.333 |
|  | Pronation | 0.981 | 0.291 | 1.000 | 0.450 | 0.978 | 0.264 | 0.895 | 0.407 |
|  | Protraction | 0.991 | 0.273 | 0.938 | 0.423 | 0.999 | 0.842 | 0.941 | 0.889 |
|  | Radial Deviation | 0.989 | 0.040 | 1.000 | 0.077 | 0.962 | 0.023 | 1.000 | 0.045 |
|  | Retraction | 0.983 | 0.348 | 1.000 | 0.516 | 0.999 | 0.929 | 1.000 | 0.963 |
|  | Rotation | 0.932 | 0.056 | 1.000 | 0.107 | 0.947 | 0.074 | 1.000 | 0.138 |
|  | Scaption | 0.992 | 0.320 | 1.000 | 0.485 | 0.967 | 0.135 | 1.000 | 0.237 |
|  | Supination | 0.966 | 0.379 | 1.000 | 0.550 | 0.960 | 0.317 | 0.988 | 0.480 |
|  | Ulnar Deviation | 0.992 | 0.000 | 0.000 | 0.000 | 0.985 | 0.030 | 1.000 | 0.058 |
|  | Vertical | 0.981 | 0.289 | 0.431 | 0.346 | 0.994 | 0.863 | 0.677 | 0.759 |
| Exercise Type | Upper Extremity Strength | 0.881 | 0.173 | 0.635 | 0.272 | 0.706 | 0.092 | 0.844 | 0.166 |
|  | Lower Extremity Strength | 0.859 | 0.407 | 0.500 | 0.449 | 0.729 | 0.236 | 0.560 | 0.332 |
|  | Trunk/Core Strength | 0.860 | 0.055 | 0.857 | 0.104 | 0.860 | 0.049 | 0.646 | 0.090 |
|  | Scapular Strength | 0.934 | 0.115 | 0.841 | 0.202 | 0.924 | 0.079 | 0.519 | 0.137 |
|  | Range of Motion (Exercise Purpose) | 0.830 | 0.208 | 0.545 | 0.301 | 0.479 | 0.085 | 0.780 | 0.153 |
|  | Flexibility/Mobility | 0.796 | 0.167 | 0.859 | 0.279 | 0.487 | 0.080 | 0.921 | 0.147 |
|  | Balance/Vestibular | 0.929 | 0.581 | 0.613 | 0.597 | 0.912 | 0.507 | 0.437 | 0.470 |
|  | Gait Training | 0.936 | 0.564 | 0.703 | 0.626 | 0.885 | 0.403 | 0.767 | 0.529 |
|  | Cardio/Aerobic | 0.991 | 0.618 | 0.783 | 0.691 | 0.984 | 0.489 | 0.643 | 0.556 |
|  | Functional Mobility | 0.762 | 0.132 | 0.651 | 0.220 | 0.605 | 0.103 | 0.794 | 0.182 |
| Exercise Purpose | Strength | 0.686 | 0.022 | 0.969 | 0.042 | 0.670 | 0.015 | 0.667 | 0.029 |
|  | Joint Mobility | 0.912 | 0.010 | 1.000 | 0.020 | 0.745 | 0.004 | 0.833 | 0.009 |
|  | Cardio | 0.996 | 0.000 | 0.000 | 0.000 | 1.000 | 0.000 | 0.000 | 0.000 |
|  | Agility | 0.989 | 0.077 | 1.000 | 0.143 | 0.997 | 0.391 | 1.000 | 0.563 |
|  | Fine Motor | 0.820 | 0.022 | 1.000 | 0.043 | 0.706 | 0.015 | 1.000 | 0.030 |
|  | Motor Control | 0.861 | 0.002 | 1.000 | 0.003 | 0.546 | 0.001 | 1.000 | 0.003 |
|  | Perception | 0.918 | 0.000 | 0.000 | 0.000 | 0.990 | 0.061 | 1.000 | 0.115 |
|  | Simulated | 0.989 | 0.541 | 0.946 | 0.688 | 0.990 | 0.524 | 0.915 | 0.667 |
| Positioning | Weight Bearing | 0.797 | 0.133 | 0.384 | 0.197 | 0.893 | 0.257 | 0.313 | 0.282 |
|  | Non-Weight Bearing | 0.761 | 0.241 | 0.344 | 0.283 | 0.841 | 0.109 | 0.023 | 0.038 |
| Average | | 0.923 | 0.272 | 0.817 | 0.354 | 0.899 | 0.335 | 0.800 | 0.374 |
